# Use of *nfsB*, encoding nitroreductase, as a reporter gene to determine the mutational spectrum of spontaneous mutations in *Neisseria gonorrhoeae*

**DOI:** 10.1186/1471-2180-9-239

**Published:** 2009-11-23

**Authors:** Daniel C Stein, Esteban Carrizosa, Stephen Dunham

**Affiliations:** 1 Department of Cell Biology and Molecular Genetics, University of Maryland, College Park, MD 20742, USA; 2 University of Pennsylvania School of Medicine, Philadelphia, PA 19104, USA; 3 Antibacterial Molecular Sciences, Pfizer Global Research and Development, Ann Arbor, MI, USA

## Abstract

**Background:**

Organisms that are sensitive to nitrofurantoin express a nitroreductase. Since bacterial resistance to this compound results primarily from mutations in the gene encoding nitroreductase, the resulting loss of function of nitroreductase results in a selectable phenotype; resistance to nitrofurantoin. We exploited this direct selection for mutation to study the frequency at which spontaneous mutations arise (transitions and transversions, insertions and deletions).

**Results:**

A nitroreductase- encoding gene was identified in the *N. gonorrhoeae *FA1090 genome by using a bioinformatic search with the deduced amino acid sequence derived from the *Escherichia coli *nitroreductase gene, *nfsB*. Cell extracts from *N. gonorrhoeae *were shown to possess nitroreductase activity, and activity was shown to be the result of NfsB. Spontaneous nitrofurantoin-resistant mutants arose at a frequency of ~3 × 10^-6 ^- 8 × 10^-8 ^among the various strains tested. The *nfsB *sequence was amplified from various nitrofurantoin-resistant mutants, and the nature of the mutations determined. Transition, transversion, insertion and deletion mutations were all readily detectable with this reporter gene.

**Conclusion:**

We found that *nfsB *is a useful reporter gene for measuring spontaneous mutation frequencies. Furthermore, we found that mutations were more likely to arise in homopolymeric runs rather than as base substitutions.

## Background

*Neisseria gonorrhoeae *(GC) is an obligate human pathogen. In order to manifest the diversity of diseases that it is able to cause, GC must produce a variety of cell surface antigens such that the appropriate antigen(s) is (are) expressed in the appropriate environment at the appropriate time. Since each of the anatomical sites that GC can infect has unique physiological properties, its success in establishing itself in a new niche requires that it rapidly adapt to its new environment. To do this, it has evolved a variety of genetic mechanisms that result in high frequency antigenic variation of its surface components. These include: intramolecular recombination for pili antigenic variation [1]; changes in the number of pentameric DNA repeat sequences for Opa expression [2]; and changes in the length of a polyguanine tract for a variety of genes, including LOS variation [3,4], pilin glycosylation [5], *pilC *expression [6] and iron utilization [7,8]. Bioinformatic analysis of the GC genome has identified a variety of additional genes that may be subject to phase variation that is mediated by some form of transient DNA mispairing [9]. Since DNA mispairings, including insertions and deletions, will arise as an intermediate in the phase variation process, and the frequency of phase variation is so high, it suggests that this pathogen should be defective in mismatch repair. However, studies in the meningococcus indicate that this organism contains a functional mismatch repair system [10], and homologs of all of the identified genes are present in the FA1090 genome [11].

In addition to the presence of a mismatch repair system, GC possesses homologs to genes that encode the proteins for recombinational repair [12], very short patch repair (DCS, unpublished observations), excision repair [13] and oxidative damage repair [14]. This indicates that GC is capable of dealing with most errors that might arise during DNA metabolism. Previous studies on GC DNA repair indicate that GC lacks error prone and photoreactivation repair systems [15,16]. Homologs to genes associated with error-prone repair and photoreactivation are not present. For complete review of DNA repair capacities, see review by Kline et. al. [11] or Ambur et. al. [17].

Nitroreductases have been identified in a wide variety of microorganisms [18-22]. They were originally studied because they are responsible for the reductive activation of certain nitro-group containing antimicrobial agents (e.g., nitrofurantoin), generating highly reactive electrophilic intermediates [23]. While the physiological role of nitroreductases in bacteria is unknown, mutants lacking nitroreductases are more resistant to nitroaromatic compounds [24]. Since the loss of gene function is associated with an increase in resistance to the antimicrobial agent, we thought that these genes might provide an ideal starting point for studying spontaneous mutation, as mutations in these genes would not be biased by the constraints of having to retain enzymatic function. We used database searches to identify a potential nitroreductase in GC, cloned and expressed the gene, verified its biochemical properties, and analyzed the DNA sequence of the gene in spontaneous nitrofurnatoin-resistant mutants.

## Methods

### Bacterial strains and growth media

*E. coli *strain DH5α-*mcr *was used for genetic manipulations and was obtained from Bethesda Research Laboratories [now Life Technologies] (Rockville, MD). *N. gonorrhoeae *strains used in this study are described in Table 1. *N. gonorrhoeae *were grown on GCK agar (GCMB, Difco supplemented with 0.5% agar and Kellogg's supplements) [25]. GCP broth was prepared by adding proteose peptone #3 (15 g), soluble starch (1 g), KH_2_PO_4 _(4 g), K_2_HPO_4 _(1 g), NaCl (5 g)/L of ultra-pure water (pH 7.5). LB agar and broth were prepared from powder obtained from US Biologicals. Plasmids used in this study are described in Table 2.

**Table 1:** Bacterial strains used in these studies

Strain	Relevant Phenotype	Source
*N. gonorrhoeae *FA1090		P. Frederick Sparling

*N. gonorrhoeae *FA19		William Shafer

*N. gonorrhoeae *F62		P. Frederick Sparling

*N. gonorrhoeae *MS11		Herman Schneider

*N. gonorrhoeae *PID2		Herman Schneider

*N. gonorrhoeae *FA1090(M1)	Spontaneous nitrofurantoin resistant mutant	This Study

*N. gonorrhoeae *FA1090 -Nfsb(mod)	Strain with a modified poly adenine tract in the beginning of the gene	This Study

*N. gonorrhoeae *FA1090 NfsB-BsmI-Σ	Strain lacking NfsB	This Study

**Table 2:** Plasmids used in these studies

Plasmids	Properties	Source
pK18	General cloning vector	[38]

pHP45Σ	Plasmid containing the Σ interposon	[39]

pNFSB	The *nfsB *region from FA1090 was amplified by PCR using primers NP1 and NP2. The amplicon was purified, digested with BamHI and cloned into the BamHI site in pK18.	This study

pEC1	The DNA between the adjacent BsmI sites were removed by digesting pEC2 with BsmI, ligating the DNA and transforming it into *E. coli*.	This study

pEC2	Two BsmI sites were inserted into pNFSB by PCR amplification using primers NfsBBsmI-3F and -2R, treating the amplicon with S1 nuclease and polynucleotide kinase, ligating the DNA and transforming it into *E. coli*.	This study

pEC3	A BsrGI site was introduced downstream of the NfsB coding sequence by PCR amplification of pEC1 using primers dwnstrm-F and dwnstrm-R. The amplicon was digested with BsrGI, ligated with a DNA fragment encoding the Σ interposon (amplified from pHP45Σ using Primer OmegaABC and digested with BsrGI) and transformed into *E. coli*.	This study

### Isolation of DNA

Chromosomal DNA for PCR reactions was prepared from bacterial cultures by resuspending a small amount of cells in 5:l 1 M NaOH. The solution was neutralized by adding 5:l of 0.5 M Tris-HCl (pH 7.5). The suspension was further diluted in 90:l of purified water, and 1:l of this solution was used as a template for PCR. Plasmid DNA isolations were carried out according to the alkaline lysis procedure [26].

### PCR

Polymerase chain reactions (PCR) were performed using various enzyme systems, based either on Taq or Pfu polymerases using chromosomal or plasmid DNA as a template. The primers used for various PCR reactions are described in Table 3. Amplification conditions were generally 41 cycles, using an annealing temperature 5°C lower than the Tm for the primer and extension times of 1-5 min. All PCR products were analyzed by agarose gel electrophoresis.

**Table 3:** Primers used in these studies

Primer Name	Primer Sequence
NP1	AAAGGATCCCATGAACGCGGATTGCAGACG

NP2	GGGGGATCCAGAAGATACCATACGCCTCT

S1	GAGATGGGTAAAATCCGGGT

S2	CGAACCGGATGCCGTAGAA

dwnstrm-F	AAAATGTACAATTTGCCGGGCGGCAGCCTGC

dwnstrm-R	AAAATGTACAGGCGTTATCTCGCTCCCGGCG

Omega-ABC	TCAGATGGCGCGCCTGTACATCGATGGTGATTGATTGACGAAGCTTTATGC

NfsB-BsmI-3F	GTTTAGGGCGCATTCAAGAACCGCAAATCGTGCCGGC

NfsB-BsmI-2R	GCGGTTCTTGAATGCGGATAGAACCTGCTCTTTGCTTAA

### DNA sequence analysis

DNA sequencing was performed by Macrogen, Inc. (Seoul, Kr.) or the DNA sequencing facility at the Center for Biosystems research at the University of Maryland. All *nfsB *sequences were obtained using Primers S1 and S2.

### Molecular biology procedures

All procedures were performed using methods described in Sambrook et al. [27]. When biological reagents were used, they were used under the conditions described by their manufacturer. Restriction enzymes, T4 DNA ligase, polynucleotide kinase and appropriate buffers were obtained from New England Biolabs (Beverly, MA). S1 nuclease was obtained from Promega (Madison, WI). DNA samples were analyzed on agarose gels (0.8-1.0%) in TBE buffer [27].

### Genetic procedures

Transformation-competent *E. coli *cells (strain DH5α-mcr) were prepared using the procedure of Inuoe [28], and stored at -80°C. To prepare cells for transformation, cells were thawed on ice, DNA added and the mixture incubated on ice for 10 min. The bacteria were heat-shocked at 37°C for 2 min., the total volume in the tube was increased to 1 ml by adding LB broth and the transformation mixture incubated at 37°C for 30 min. to 1 hr. to allow the bacteria to recover and begin expressing antibiotic-resistance proteins. Transformed bacteria were plated onto LB agar plates containing appropriate antibiotics and, if necessary, X-gal.

For transformation of *N. gonorrhoeae*, piliated bacteria were resuspended to light turbidity in 1 ml GCK+ 10 mM MgCl_2 _+ Kellogg's supplement + 0.42% NaHCO_3_. DNA was added to the culture, and the bacteria were incubated at 37°C with shaking for 2-6 hours. Bacteria were plated onto GCK agar plates containing the appropriate antibiotic, and the plates incubated for 36-48 hrs. When transformations were performed under nonselective conditions, a spot transformation procedure was used [29]. For transformation, two piliated colonies were resuspended in 100:l GCP + 200 mM MgCl_2 _+ 0.42% NaHCO_3 _+ Kellogg's supplement. The cell suspension was diluted 1:10, and additional two-fold serial dilutions were then carried out 9 times. An aliquot (5:l) of each suspension was spotted onto a GCK agar plate. To each spot, 5:l of DNA were added. After incubation overnight at 37°C with 4% CO_2_, individual colonies were isolated and streaked for isolation on GCK agar plates. The next day, individual colonies were inoculated onto GCK and spectinomycin-containing GCK agar plates. This procedure was repeated until spectinomycin-sensitive colonies were obtained. The correct replacement of the desired DNA fragment by the transformation process was verified by PCR amplification of the desired region, and restriction digestion analysis of the PCR amplicon, or direct DNA sequencing of the PCR amplicon.

### Sequence modification of *nfsB*

The *nfsB *gene from strain FA1090 was amplified by PCR using primers NP1 and NP2. The amplicon was purified, digested with BamHI and cloned into the BamHI site in pK18, resulting in plasmid pNFSB. To alter the poly adenine sequence at the 5' end of the gene from AAAAA to AAGAA, PCR primers NfsB-BsmI-F and NfsB-BsmI-R were designed. The resulting amplicon was digested with BsmI, ligated, and introduced into *E. coli *by transformation, giving pEC1. Plasmid pEC1 was amplified via PCR using the primers dwnstrm-F and dwnstrm-R, allowing for the insertion of a BsrGI site. A spectinomycin resistance cassette was amplified from pMP45Σ using primer Omega-ABC, and ligated into the BsrG1 site, resulting in pEC3. This plasmid was used to transform strain FA1090 to spectinomycin resistance, resulting in strain NfsB-BsmI-Ω. The spectinomycin resistance cassette was removed using the spot transformation procedure [29] with pEC1, producing a strain that had an intact modified *nfsB *gene(FA1090-NfsB(mod)). The correct construction was verified by DNA sequence analysis of a PCR amplicon. The DNA sequences for *nfsB *from the various strains have been submitted to GenBank with the following accession numbers: F62, GU112780; MS11, GU112781; FA19, GU112782; and PID2, GU112783. Point mutations in *nfsB *that resulted in nitrofurantoin resistance are identified in GenBank as accession numbers: GU112770; GU112771; GU112772; GU112773; GU112774; GU112775; GU112776; GU112777; GU112778; and GU112779.

### MIC determinations

The minimum inhibitory concentration (MIC) of nitrofurantoin for several gonococcal strains was determined by a plate dilution method. Approximately 10^8 ^gonococci were spotted onto media containing various amounts of nitrofurantoin (1 μg/ml, 2 μg/ml, 3 μg/ml, 4 μg/ml, 6 μg/ml, 8 μg/ml, 16 μg/ml and 32 μg/ml). The inoculated plates were incubated overnight, and the MIC was defined as the amount of nitrofurantoin needed in the plate to completely inhibit the growth of the organisms in 24 hours.

### Spontaneous mutation frequency determination

Cultures of GC were grown in GCP broth + 0.42% NaHCO_3 _and Kellogg's supplement to exponential growth phase, and aliquots (~1 × 10^8 ^cfu) plated onto GCK plates containing 3:g/ml nitrofurantoin. Viable counts were determined by plating cells onto GCK agar plates. Mutation frequencies were defined as the number of colonies obtained on nitrofurantoin-containing media divided by the number of colonies obtained on GCK media.

### Nitroreductase assay

Nitroreductase activity was measured by a modification of the method of Whiteway et al. [24]. Cultures (100 ml) of GC were grown in GCP broth + 0.42% NaHCO_3 _and Kellogg's supplement at 37°C with shaking to a turbidity of 100 klett units. Cells were collected by centrifugation (~4,000 rpm for 10 min in a Sorvall GSA rotor), washed with PBS, and resuspended in 5 ml 100 mM Tris-HCl, pH 7.5. Cells were lysed by sonication using a Branson sonicator with the microprobe, set on full power, using 5 10 sec pulses (Suspensions were incubated on ice for 1 min between pulses). The sonicates were clarified by centrifugation (~10,000 rpm for 30 min in a Sorvall SS-34 rotor) and the supernatants collected. Protein concentrations of each sample were measured with the BioRad protein assay (Hercules, CA) using BSA as a standard, and samples were normalized to the same protein concentration in 100 mM Tris-HCl, pH 7.5. Samples containing 800:l lysate and 0.1 mM nitrofurazone were placed in a quartz cuvette, and the reaction initiated by adding 100:l NADPH (2 mM stock). A control reaction was performed using water instead of nitrofurazone. Reactions were incubated at room temperature and absorbance was measured every 30 sec at 400 nm.

### Bioinformatics

A homolog of *E. coli nfsB *in the gonococcus was identified by submitting the entire *E. coli nfsB *protein sequence to http://blast.ncbi.nlm.nih.gov/Blast.cgi using the tblastn program. The database option was set to "nucleotide collection," and limited to *Neisseria gonorrhoeae*. The database option was set to "bacteria," and the number of best-scoring sequences to show was set to 250. The top scoring hits from unique genera were aligned using ClustalW http://www.ebi.ac.uk/Tools/clustalw/.

## Results

### MIC/Spontaneous Mutation Frequency Studies

**Figure 1:**
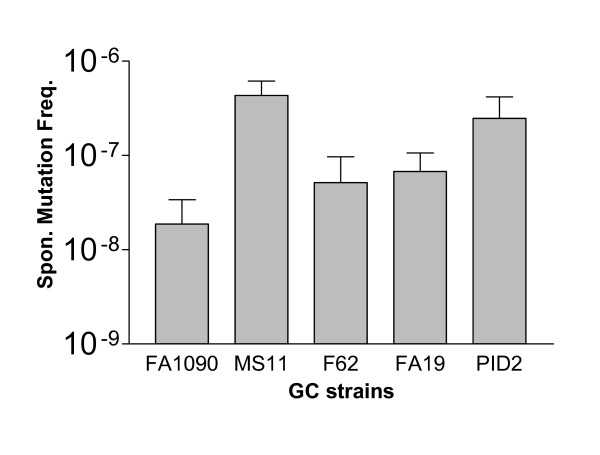
**Spontaneous mutation frequency of various lab strains of *N. gonorrhoeae***. Mutation frequencies were determined by counting the number of colonies arising on the GCK + Nitrofurantoin (3 μg/mL) plates after 48 hours of incubation at 37°C, 6% CO_2_, and dividing this number by the number of colonies arising on the GCK plates after 48 hours of incubation at 37°C, 6% CO_2_. Data represents experiments done in triplicate, with error bars representing standard error.

If *N. gonorrhoeae *possesses a nitroreductase, it should be sensitive to antimicrobial agents that are activated by nitroreductases and it should be possible to isolate mutants that become resistant to these activated antimicrobials due to the organism's loss of nitroreductase activity. We determined the MIC for nitrofurantoin, an antimicrobial agent that is activated by nitroreductases, for several different gonococcal strains. Plates were analyzed after 24 hrs incubation, and the concentration of nitrofurantoin that completely inhibited the ability of GC to form visible colonies was determined. The data indicated that the MIC for nitrofurantoin was approximately 3:g/ml for all strains tested (data not shown). When plates were incubated for an additional 24 hrs, a small number of colonies arose, and these were presumptive nitrofurantoin-resistant mutants. By comparing the number of colonies found on an agar plate after 48 hours incubation in the presence of nitrofurantoin to the number of colonies obtained when a similar aliquot was inoculated onto media lacking the antimicrobial agent, we were able to calculate the spontaneous mutation frequency to resistance to this agent. The data (Fig. 1) indicate that the mutation frequency associated with this antimicrobial agent varied about 10 fold among strains, with FA1090 being the least mutable among the strains tested, and MS11 being the most mutable. However, since it was possible to isolate mutants that readily grew on media containing levels of nitrofurantoin above the MIC, we hypothesized that the mutation responsible for this phenotype was in the coding sequence for the putative gonococcal nitroreductase gene.

### Identification of potential nitroreductase genes

*E. coli *possesses two nitroreductases that can reduce these nitro-aromatic compounds; *nfsA *and *nfsB*, plus a nitroreductase activity encoded by a gene that has yet to be identified [18,24]. Therefore, it is possible that GC may possess additional genes that confer nitroreductase activity. In *E. coli*, resistance to these nitro-aromatic antimicrobial agents occurs in a step-wise manner. A mutation that knocks out the function of NfsA raises the MIC about three fold. A second mutation that knocks out the function of NfsB increases resistance to about 10 times the MIC of wild-type strains [18,24]. All attempts to isolate second-step mutants in *N. gonorrhoeae *were unsuccessful, indicating that this species only contains a single functional nitroreductase, or that the additional nitroreductases were insensitive to nitrofurantoin.

**Figure 2:**
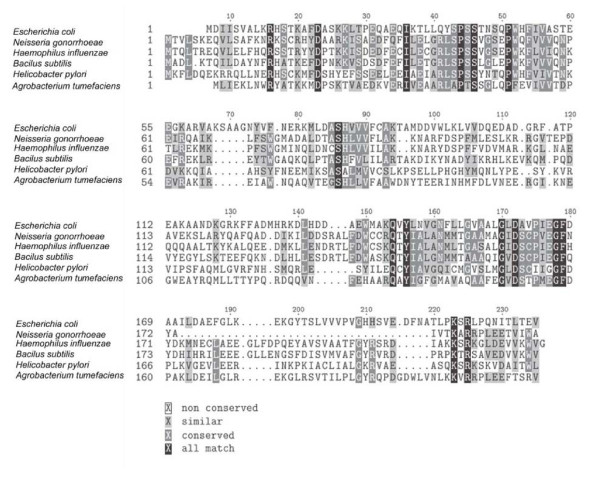
**Sequence similarities of nitroreductases**. The amino acid sequence encoding various nitroreductases were aligned. Identical residues are highlighted in black, and conserved substitutions are highlighted in grey. The sequences represent (Bacterium, Genebank identification number): *Escherichia coli *NP_415110.1, *N. gonorrhoeae *FA1090, NC002946; *Haemophilus influenzae*, Q57431; *Bacillus subtilis*; O34475; *Helicobacter pylori*, NP459570; and *Agrobacterium tumefaciens *str. C58, NP534964.

Since two nitroreductases have been identified in *E. coli*, *nfsA *and *nfsB *[30,31], we used the amino acid sequence for these two gene products to search the gonococcal translated genomic DNA sequence database. No significant similarity was found to *nfsA*. However, an ORF encoding a protein with some similarity to *nfsB *was found. This gonococcal sequence possessed 25% identity and 44% similarity to the *E. coli *NfsB protein, suggesting that this gene encoded a nitroreductase. The amino acid sequence of the gonococcal homolog was used to probe the GenBank database, and ORFs that possessed significant similarity to it were identified. The data presented in Figure 2 is an alignment of proteins that possessed significant similarity to the gonococcal *nfsB *homolog. All of these proteins have nitroreductase activity.

### DNA sequence analysis of *nfsB *from various gonococcal strains

The *nfsB *DNA sequence for *N. gonorrhoeae *strains F62, FA19, MS11, and PID2 was determined by sequencing PCR products amplified from their respective chromosomes. Sequence data were derived from multiple independent amplicons. The data indicated that the DNA sequence was highly conserved as all sequences obtained were identical to the *nfsB *DNA sequence of FA1090, with the exception of strain PID2. This strain possessed a single nucleotide polymorphism (using the adenine of the start codon as base one, at base 575 from the start codon, this base is a guanine in FA1090 and a cytosine in PID2) that would result in an amino acid substitution in NfsB at residue 192 (a glycine in FA1090 and an alanine in PID2). Since these proteins were essentially identical, it suggests that the variability in spontaneous mutation frequencies observed in these strains could reflect different DNA repair capacities for the various strains.

### Nitroreductase activity in *N. gonorrhoeae*

**Figure 3:**
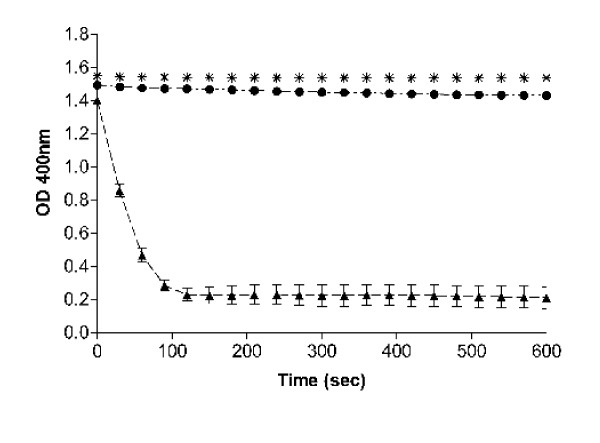
**Nitroreductase activity in *N. gonorrhoeae *strains**. Cell sonicates were tested for their ability to produce a loss of absorbance at 400 nm, indicating a reduction of nitrofurazone by an active nitroreductase. The symbols represent: FA1090 (□); FA1090 extract lacking NADPH (□); and FA1090(M1), an *nfsB *mutant of FA1090 (□). Samples measured every 30 sec for 10 min. The data represents the average of 7 separate experiments with the error bars representing the standard error.

A spectrophotometric assay was performed to measure nitroreductase activity in GC. Lysates from wild type and nitrofurantoin resistant mutants were assayed for nitroreductase activity using a spectrophotometric assay that detects the loss of the substrate, nitrofurazone, using a method adapted from Whiteway et al. [24]. The data (Fig. 3) show that we were able to detect nitroreductase activity from strain FA1090, but that a spontaneous nitrofurantoin-resistant mutant (FA1090(M1)) lacked any detectable nitroreductase activity.

### Genetic basis of nitrofurantoin resistance

In order to verify that the nitrofurantoin resistance observed in the mutant was due to alterations in *nfsB*, the DNA sequence of this region was determined from spontaneous nitrofurantoin resistant mutants by amplifying the *nfsB *region from this strain. The data indicate that FA1090(M1) possessed a small insertion of 7 nucleotides about midway through the coding sequence, producing a frame shift mutation in *nfsB*. This genetic data supported the hypothesis that the nitrofurantoin resistant phenotype is due to the loss of nitroreductase activity. Conclusive evidence that this gene was responsible for nitrofurantoin resistance was obtained by deleting the coding sequence for this gene from FA1090 and then demonstrating that FA1090NfsB-BsmIS lacked nitroreductase activity (data not shown).

### Identification of the genetic basis of spontaneous nitrofurantoin resistant mutants

We isolated numerous independent spontaneous nitrofurantoin resistant mutants and determined the DNA sequence of the *nfsB *gene in these strains. Most of these mutants (90%) possessed the insertion of an adenine in a run of 5 adenines near the beginning of the gene, suggesting a bias for base insertion during DNA replication at this position. This gene contains three other polynucleotide runs of five nucleotides distal to the start codon; 2 poly adenines and one polythymine. Interestingly, even though we were able to isolate base insertions at each of these three clusters, none of the clusters showed the elevated propensity for generating spontaneous mutations.

**Figure 4:**
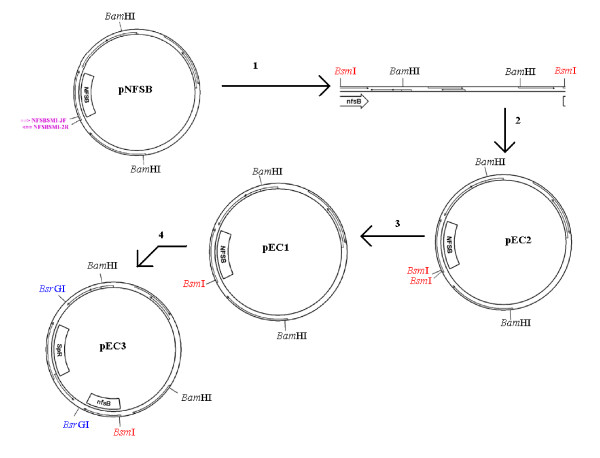
**Schematic illustrating the strategy used to modify the *nfsB *coding region**. Each numbered arrow corresponds to the procedures summarized below: 1: PCR using primers NfsBsmI-3F and NfsBsmI-2R to introduce a *Bsm*I recognition sequence and to alter a poly-A tract. 2: Treatment with S1 nuclease to create blunt ends, polynucleotide kinase to phosphorylate 5' ends, and T4 DNA ligase. *E. coli *were transformed using this construct (pEC2). Plasmid DNA was isolated by alkaline lysis. 3: Treatment with *Bsm*I to generate pEC1. Digestion product was ligated with T4 DNA ligase. The construct was transformed into *E. coli*. 4: pEC1 was amplified with primers dwnstrm-F and dwnstrm-R. The product was ligated to the omega fragment, a PCR product of pHP45Σ with the OmegaABC primer, to generate pEC3. The omega fragment is symmetric, so one primer amplifies in both directions

To eliminate the bias introduced by the 5 bp polyadenine run at the 5' end of the gene, this DNA sequence was altered to remove the poly-A tract, while preserving the corresponding amino acid sequence. The plasmid, pEC3 was constructed as described in figure 4. Plasmid DNA was isolated from *E. coli *and DNA used to transform *N. gonorrhoeae*. Spectinomycin resistant transformants were identified, and DNA sequence analysis of a PCR amplicon derived from the constructed strain indicated that the derivative of FA1090, FA1090-NfsB(mod) contained the desired sequence modification. Nitroreductase assays of this strain indicated that it possessed wild-type NfsB activity (data not shown).

We isolated spontaneous nitrofurantoin resistant mutants of strain FA1090-NfsB(Mod), by plating this strain on GCK agar containing 3:g/ml nitrofurantoin. We determined the genetic basis of 107 individual independently isolated mutants that arose from this plating by amplifying the desired region using Primers NP1 and NP2, and determining the DNA sequence of *nfsB *using Primers S1 and S2. The experimental design employed should allow for the identification of six different types of mutations, four that would be manifested within the coding sequence of *nfsB *(missense mutations, nonsense mutations, insertions, deletions) and mutations outside of the coding sequence, presumably mutations that effected nitrofurantoin uptake, or in the regulation of *nfsB *expression. The data presented in Table 4 summarizes the types of mutations identified by our DNA sequence analysis of PCR amplicons. The data indicate that about half of the mutants possessed point mutations, one quarter possessed insertions and one quarter possessed deletions. The largest insertion mutant was 7 bp in length and the largest deletion was 5 bp in length. None of the multiple base insertions appeared to be the result of duplications in the native coding sequence and none of the deletions appeared to eliminate repeated sequences or sequences that contained obvious secondary structure. Furthermore, insertions did not seem to show a preference for expanding short (4 bp) polynucleotide runs, but seemed to randomly incorporate one or more nucleotides.

**Table 4:** Analysis of mutations resulting in nitrofurantoin resistance

Point mutations^a^				Frameshift mutation				
**Nonsense**		**Missense**		**Insertions (single site)**		**Deletions (single site)**		

CAA->TAA	7	Transitions		Single base	22	Single base	16	

CAG->TAG	11	C->T	5	Multiple bases	4	Multiple bases	9	

TCG->TAG	9	T->C	2					

GAG->TAG	5	A->G	0					

TGG->TGA	1	G->A	1					

		Transversions			Mutations in promoter region			3

		T->A	3					

		A->T	0					

		G->C	1					

		C->G	1					

		T->G	5					

		G->T	0					

		A->C	0					

		C->A	2					

Total:	33		20		26		25	3

^a^Of the 53 point mutations examined, 27 were transitions and 26 were transversions.

### Use of nonsense mutations to characterize transition and transversion rates

Any point mutation that is capable of generating a stop codon could generate a cell that is resistant to the killing action of nitrofurantoin. Visual analysis of the coding sequence for *nfsB *identified 23 possible bases where a single base change would result in the production of a stop codon. We identified 33 mutations that resulted from this type of base change. The distribution of the mutations obtained suggested that no hot spot for mutation existed in any of these sequences (see Table 4). We obtained a total of 27 transition mutations and 26 transversion mutations, suggesting that no preference exists for generating these types of mutations. Interestingly, the number of deletion and insertion mutations occurred at approximately the same frequency as the number of transition and transversions.

### Analysis of mutations

**Figure 5:**
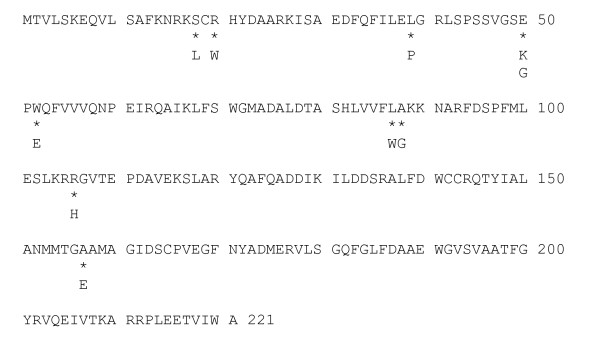
**Mutations in *nfsB *resulting in nitrofurantoin resistance**. Missense mutations were identified at 9 different sites throughout the *nfsB *coding region. Residues affected by missense mutations are marked by *, and the altered amino acid is shown below.

While the majority of the collected mutations were insertion, deletion or nonsense mutations, we did identify a variety of key residues in the NfsB protein that are essential for its function. The data in Figure 5 indicate key residues, that when mutated, resulted in the loss of sensitivity to nitrofurantoin. While we did not perform biochemical analysis on the nitroreductase of all of these mutants, of those tested, we detected no activity, suggesting that these mutations reside in key residues.

## Discussion

Phase variation is a reversible, high-frequency phenotypic switching that is mediated by changes in the DNA sequence that effects the expression of the target gene. The ability of individual genes to phase vary contributes to population diversity and is important in niche adaptation. Understanding which genes are capable of undergoing phase variation is the first step defining which genes are important in disease pathogenesis. Being able to determine the rate at which these processes occur and the nature of any factors that influence them is integral to understanding the impact of these processes on the evolution and dynamics of the population as a whole and on the host-bacterium interaction. Studies on phase variation in the gonococcus have been hampered by our lack of knowledge of background mutation frequencies.

We reasoned that analysis of genes, whose loss of function would provide for a positive selection, would allow for an unbiased comparative analysis of spontaneous mutations, and the study of spontaneous mutation in these genes would provide baseline information for future studies on factors that might effect antigenic variation. We further reasoned that with this knowledge, we could distinguish between changes in gene expression that were the result of slip strand mispairing during DNA replication from changes due to other forms of mistakes that occur during DNA replication.

We determined that *N. gonorrhoeae *encodes a nitroreductase gene (*nfsB*). The inability to isolate second-step nitrofurantoin resistant mutants suggested that the gonococcus only contained a single nitroreductase. We obtained biochemical data to support this conclusion, where mutants that were resistant to nitrofurantoin lost the ability to reduce nitrofurantoin. Since cell lysates that did not contain the co-factor NADPH had no nitroreductase activity, it indicated an absolute requirement for this co-factor. However, we did not determine if other molecules such as NADH could substitute for the NADPH requirement, or if the organism might have other nitroreductases that utilized a cofactor other than NADPH.

We determined the nature of spontaneous mutation by analyzing where mutations occurred in *nfsB*. While we were able to identify mutations that would result in amino acid substitutions in the region involved in FMN binding [24], the majority of the mutations were outside of this region, with most of them clustering in the amino terminus of the protein. This was somewhat surprising, given that this region of the protein is not well conserved in known nitroreductases.

The results of the spontaneous mutation frequency plating experiments and the subsequent genetic analysis showed that nitrofurantoin resistance is a potential target for analyzing mutation in the gonococcus. The fact that almost all mutations originally examined resulted in an extension of a polyadenine run of 5 adenines was surprising, as it is thought that this sequence is too short to participate in strand slippage. Furthermore, the absence of slippage at two other polyadenine runs of 5 in other locations indicates that sequence context is important in strand slippage.

The use of *nfsB *as a reporter system allowed us to assess the nature of spontaneous mutation in an unbiased fashion. If one removes the high frequency of errors that occurred in the polynucleotide run of adenines, the propensity of errors directed towards transitions and transversions occurred at a similar frequency to insertion or deletion mutations. However, the high rate of insertions and deletions is in contrast to what was observed by Schaaper and Dunn [32], who in their studies of spontaneous mutation in the *lacI *gene of *Escherichia coli *saw that single base insertions and deletions only made up 4.2% of their observed mutations. While we observed that single base insertions and deletions accounted for ~40% of our observed mutations in a background where a run of five adenines was removed, if the bias observed at this sequence was included, insertions would have made up about 75% of all observed mutations. The implication of this finding would suggest that homopolymeric runs should have a tendency to increase, and that they should dominate the types of mutations seen in the gonococcus. This is precisely what is observed. The mechanism by which gonococcal DNA polymerase allows this to occur, and the inability of the gonococcus to efficiently correct insertions indicates that gonococcal DNA repair is somewhat different from that seen in *E. coli*.

Most of our understanding of DNA repair in the Neisseria has come from studies focused on understanding the contribution of various DNA repair proteins in preventing mutations in *rpoB *in the gonococcus or meningococcus. These studies have analyzed numerous strains for the rate of spontaneous resistance to rifampicin, and find that in general, this rate is between ~1 × 10^-8 ^- 1 × 10^-9 ^[33-36]. Our data indicate that mutants resistant to nitrofurantoin arose at a much higher frequency (~3 × 10^-6 ^- 8 × 10^-8^). We believe that the higher mutation frequencies that we observed relate to the nature of the selection procedure employed. Mutation screens designed to detect *rpoB *mutants are constrained in that they must result in the production of a functional protein. Our screening procedure allowed us to detect any mutation that results in the loss of function of the target, and hence is able to identify insertions and deletions, as well as point mutations. We believe that the elevated mutation frequency that we observed for *nfsB*, relative to that observed by others for *rpoB *was due to the presence of the polyadenine sequence in *nfsB *and our ability to detect frame shift mutations.

Race and coworkers [37] have solved the crystal structure of NfsB isolated from *E. coli*. Interestingly, none of the mutations that we identified were contained in any of the key residues that they demonstrated to be interacting with nitrofurantoin. However, a significant number of the amino acid substitutions that we identified would be expected to have dramatic structural implications.

## Conclusion

In summary, we found that *nfsB *is a useful reporter for measuring spontaneous mutation frequencies. Its ability to detect elevated mutation frequencies in very short polynucleotide runs indicates that any gene that contains a short polynucleotide run has the potential to phase vary.

## Authors' contributions

SD and DS conceived of the scientific concept that formed the basis of this manuscript. EC performed the experiments and participated in the data analysis. DS wrote the manuscript.
